# Assessing the Response of Ecosystem Water Use Efficiency to Drought During and after Drought Events across Central Asia

**DOI:** 10.3390/s20030581

**Published:** 2020-01-21

**Authors:** Jie Zou, Jianli Ding, Martin Welp, Shuai Huang, Bohua Liu

**Affiliations:** 1 Xinjiang Common University Key Lab of Smart City and Environmental Stimulation, College of Resources and Environment Sciences, Xinjiang University, Urumqi 830046, China; zoujiexj@163.com (J.Z.); 107556516063@stu.xju.edu.cn (S.H.); 107556517070@stu.xju.edu.cn (B.L.); 2 Key Laboratory of Oasis Ecology under Ministry of Education, Xinjiang University, Urumqi 830046, China; 3 Faculty of Forest and Environment, Eberswalde University for Sustainable Development, Eberswalde 16225, Germany; martin.welp@hnee.de

**Keywords:** water use efficiency, drought, arid land, legacy effect, resilience

## Abstract

The frequency and intensity of drought are expected to increase worldwide in the future. However, it is still unclear how ecosystems respond to drought. Ecosystem water use efficiency (WUE) is an essential ecological index used to measure the global carbon–water cycles, and is defined as the carbon absorbed per unit of water lost by the ecosystem. In this study, we applied gross primary productivity (GPP), evapotranspiration (ET), land surface temperature (LST), and normalized difference vegetation index (NDVI) data to calculate the WUE and drought index (temperature vegetation dryness index (TVDI)), all of which were retrieved from moderate resolution imaging spectroradiometer (MODIS) data. We compared the mean WUE across different vegetation types, drought classifications, and countries. The temporal and spatial changes in WUE and drought were analyzed. The correlation between drought and WUE was calculated and compared across different vegetation types, and the differences in WUE between drought and post-drought periods were compared. The results showed that (1) ecosystems with a low (high) productivity had a high (low) WUE, and the mean ecosystem WUE of Central Asia showed vast differences across various drought levels, countries, and vegetation types. (2) The WUE in Central Asia exhibited an increasing trend from 2000 to 2014, and Central Asia experienced both drought (from 2000 to 2010) and post-drought (from 2011 to 2014) periods. (3) The WUE showed a negative correlation with drought during the drought period, and an obvious drought legacy effect was found, in which severe drought affected the ecosystem WUE over the following two years, while a positive correlation between WUE and drought was found in the post-drought period. (4) A significant increase in ecosystem WUE was found after drought, which revealed that arid ecosystems exhibit high resilience to drought stress. Our results can provide a specific reference for understanding how ecosystems will respond to climate change.

## 1. Introduction

Drought is a natural event characterized by a supply of natural water that does not meet existing needs, or water that is below normal water levels for a long time [1]. As reported by the Intergovernmental Panel on Climate Change (IPCC), the frequency and intensity of global drought events will increase in the 21st century under the context of global climate change, due to global land surface temperature (LST) increases and precipitation decreases. As an important part of the global carbon–water cycle, ecosystem plants absorb carbon dioxide from the atmosphere through photosynthesis and lose water through transpiration, and these plants are important drivers of the energy and water cycles between the atmosphere and terrestrial ecosystems [2]. Drought is the main factor affecting the carbon–water cycle of terrestrial ecosystems, as it increases the individual mortality of vegetation; inhibits vegetation growth; and triggers forest fires, forest pests, and biological invasions [3,4,5,6]. Therefore, an increasing number of scholars have begun to study the relationship between drought and ecosystems, and the response mechanism of ecosystems to drought.

Ecosystem water use efficiency (WUE) is an important indicator of the coupling of carbon and water cycles, and WUE is often employed to characterize ecosystem carbon and water cycle indicators. The WUE is defined as the ratio of carbon absorption to water loss. The WUE links biological processes (i.e., plant leaf photosynthesis and transpiration) to physical processes on the land surface (i.e., evaporation) [7]. Several global and regional studies have been carried throughout the world [8,9], and have shown that the spatiotemporal distribution of the WUE is affected by many factors, such as latitude, surface temperature, and plant species; however, water availability (hydrological conditions) is the main factor affecting the spatial and temporal distributions of WUE through changes to the global water balance and water availability [10,11]. Therefore, research on the spatial and temporal distribution of drought and the WUE plays an important role in understanding the impacts of climate change in terrestrial ecosystems.

The response of the WUE in an ecosystem to drought has become a popular topic because of climate change. In general, a common mechanism is that the WUE will increase in water-deficient or arid environments at the vegetation leaf and observation site scale, as the leaf stomatal conductance will decrease under drought conditions, resulting in decreased water loss. This regulation of stomatal behavior is widely used in ecosystem models [12,13]. However, studies have shown that under severe drought conditions, the WUE decreases rapidly at the leaf scale, because severe drought causes plant activity losses, which changes the effects of drought on vegetation from stomatal limitation to nonstomatal limitation. However, this relationship between WUE and drought has not been applied in ecosystems modeled under severe drought conditions [14,15,16]. In recent studies, the relationship between ecosystem WUE and drought showed a large divergence across different ecosystems [6]. Therefore, there is an urgent need to improve the knowledge to explain and understand the relationship between WUE and drought, especially under severe drought conditions.

An improved understanding of the effects of drought on ecosystems can be obtained by understanding the changes in ecosystems before and after drought. In general, ecosystems exhibit a certain degree of resilience after a drought, that is, the ability of an ecosystem to return to its predrought state [17]. For example, in grasslands, drought can rapidly reduce the aboveground biomass of grassland vegetation, but the belowground parts are rarely affected, which serve as preparations for post-drought recovery [18,19]. Many trees have a resprouting ability and are able to return to their pre-drought state [20,21]. However, severe drought may lead to vegetation death as a result of the threshold of ecosystem resilience being exceeded and changes to different ecological processes at various spatiotemporal scales, such as tree regeneration and growth and ecosystem productivity. In addition, there is a body of empirical and theoretical evidence that sustained drought can cause herbaceous vegetation losses and increase bare land areas, which may trigger the arid and semiarid ecosystems to switch to an alternative steady state, which is characterized by low water infiltration and high runoff [22]. The results of these studies indicated that if there is no human intervention in the plant community, it will be impossible to rebuild to the previous state [23]. Therefore, it is necessary to study the changes in ecosystem states before and after drought, which can help us understand ecosystem processes under drought disturbances.

In previous studies, scholars have defined several ways to calculate WUE based on the definition of ecosystem WUE. Among these methods, ecosystem carbon sequestration is often determined using several indicators, including net primary productivity (NPP) [24], gross primary productivity (GPP) [4,14], aboveground NPP (ANPP), and net ecosystem productivity (NEP) [25], of which GPP is the most used to characterize carbon absorption of ecosystems. At the leaf scale, water loss is characterized by the leaf evaporation rate. Theoretically, the leaf evaporation rate at the leaf scale should be used to determine the water loss of an ecosystem; however, because of measurement difficulties, evapotranspiration (ET) is often applied to indicate water loss [26]. In this study, we use the ratio of GPP to ET to define WUE, which is the most widely used method in most studies. There are three ways to obtain measurements of ecosystem WUE, namely, eddy covariance (EC), process-oriented ecosystem models [4,6,27,28], and remote sensing measurements [29]. Of these three methods, EC measurements have the highest accuracy, but applications are limited by its small scale [30,31]. Modeling methods can provide long-term and large-scale WUE estimates, but errors caused by model uncertainty are a problem. In recent years, an increasing number of studies have used remote sensing data to calculate WUE. On the one hand, the WUE obtained via remote sensing exhibits a high correlation with the WUE measured by EC, and has been verified in both global and region studies [4]. On the other hand, remote sensing data can solve problems related to the finite scale of EC observations and the errors of ecological model simulations [31,32]. Therefore, the WUE in this study is calculated by GPP/ET based on remote sensing data.

The temperature vegetation dryness index (TVDI) has been applied as a very popular drought index in many studies [33,34,35], and the accuracy of the TVDI has been verified in different areas and different vegetation landscapes. Przeździecki, et al. [36] constructed a TVDI model using the triangular method from moderate resolution imaging spectroradiometer (MODIS) satellite images over two states of the United States, and the result showed a reasonable relationship between the TVDI and the soil moisture content at different depths. Holzman, et al. [37] compared the relationship between the TVDI calculated from the MODIS data and the underground soil moisture of farmland and native grassland in Argentina. A high correlation was found (R^2^ > 0.69), indicating that the combination of surface temperature and reflectance data can be used to monitor the underground soil moisture in vegetation areas. Dorjsuren, et al. [38] investigated the drought characteristics in arid regions of Mongolia using MODIS satellite remote sensing images. The comparison of the drought index and the soil moisture content at a depth of 10 cm revealed a correlation coefficient of 0.74. Therefore, previous studies have shown that the TVDI based on MODIS satellite remote sensing data can adequately monitor and quantify the impacts of drought on ecosystems. In this study, we chose the TVDI based on MODIS data as the drought index, as MODIS data can maintain a spatial resolution consistent with that of the ecosystem WUE.

Central Asia is one of the largest arid regions in the world, and includes Kazakhstan (KAZ), Turkmenistan (TKM), Uzbekistan (UZB), Kyrgyzstan (KGZ), Tajikistan (TJK), and the Xinjiang Uygur Autonomous Region of China (CHN) [39]. In recent years, climate change and frequent human activities have caused tremendous changes in Central Asia. A recent study showed that increasing temperature and decreasing precipitation on a global scale led to a severe drought in Central Asia, and these changes will potentially impact vegetation growth and ecosystems in Central Asia [40]. However, this condition also provides an opportunity to study the response mechanisms of ecosystems to drought. In this study, MODIS GPP and ET measurements were used to calculate the WUE, and the relationships between ecosystem WUE and drought were examined in Central Asia from 2000 to 2014. The objectives of our study were as follows: (1) calculate and compare the mean WUE values from 2000 to 2014 at different drought levels, biome types, and countries; (2) calculate the spatiotemporal patterns of WUE and drought characteristics; (3) quantify the relationship between WUE and the drought index in drought and post-drought periods and attempt to determine the potential impact of drought on ecosystems; and (4) evaluate the response of WUE to variations in dry–wet conditions after drought.

## 2. Methods

### 2.1. Data Sources

GPP and ET data from 2000 to 2014 were both retrieved from MODIS, which were produced by the Numerical Terra dynamic Simulation Group (http://www.ntsg.umt.edu). The MODIS GPP product (MOD17A3) was developed based on a light-use efficiency model [41]. The accuracy and reliability of this model have been validated in many regions [25,42,43]. MOD17A3 has been widely used in studies on regional or global ecosystem carbon cycles [44]. The global MODIS ET product (MOD16A3) was estimated based on the Penman–Monteith model, which uses meteorological reanalysis data and vegetation property dynamics (e.g., land cover, leaf area index, and albedo) retrieved from MODIS as input variables [45,46]. Validations of this product using flux tower data from stations in the USA [47], Asia [48], and other regions have indicated reasonable accuracy [14]. We used the land cover type CMG product (MCD12C1) to analyze the observed changes in the selected datasets. The Central Asian study region is primarily represented by the following five land cover classes: grasslands (26.91%), barren land (24.61%), croplands (9.34%), forest (13.21%), and shrublands (11%). We did not analyze the barren pixels because there were no ET data in barren pixels.

### 2.2. Drought Index

The study conducted by Sandholt (2002) found that there were many isoclines in the LST-NDVI feature space; they simplified these feature space into a triangle shape and proposed the TVDI [34]. The TVDI is calculated using Equation (1).

(1) $$TVDI=\frac{LST-LST_{min}}{LST_{max-LST_{min}}}$$

where LST is the land surface temperature of the observed pixel, and $LST_{max}$ is the maximum observation temperature corresponding to the NDVI value of the observed pixel, that is, the dry edge, as follows:

(2) $$LST_{max}=a+b\times NDVI$$

$LST_{min}$ is the minimum observation temperature corresponding to the NDVI value of a specific pixel, the wet edge, as follows:

(3) $$LST_{min}=c+d\times NDVI$$

NDVI is the normalized difference vegetation index of the observed pixel; *a, b, c,* and *d* are the coefficients of the linear dry- and wet-edge linear fitting equations, respectively. The TVDI values range from 0 to 1, where TVDI = 1 indicates that the soil has no evaporation or a limited moisture supply, and TVDI = 0 indicates that the soil has maximum evaporation or unlimited moisture supply. The drought was divided into five grades by using the method of dividing the drought level in arid and semiarid areas, as follows: humid (0 ≤ TVDI < 0.2), normal (0.2 ≤ TVDI < 0.4), slight drought (0.4 ≤ TVDI < 0.6), drought (0.6 ≤ TVDI < 0.8), and severe drought (0.8 ≤ TVDI < 1) [49].

### 2.3. Trend Analysis, Correlation Analysis, and Regression Model Establishment

The Mann–Kendall trend analysis was used to determine the trends of WUE and drought over the period of 2000–2014 [50]. For the Mann–Kendall non-parametric test method, the advantage is that the data series does not require normality. This method is widely applied in trend studies of climate data and vegetation data [51,52]. The significance level was assessed at *p* < 0.05. The relationships between ecosystem WUE and TVDI over the period of 2000–2014 were examined using linear correlation analysis.

To investigate the legacy effect of drought on WUE, we built three linear regression models between WUE and the TVDI in different years.

(4) $$WUE_{current}=a\times TVDI_{current}+b$$

(5) $$WUE_{current}=a\times TVDI_{current}+b\times TVDI_{previous\;one\;year}+c$$

(6) $$WUE_{current}=a\times TVDI_{current}+b\times TVDI_{previous\;one\;year}+c\times TVDI_{previous\;two\;year}+d$$

where $WUE_{current}$ is the current year WUE, and $TVDI_{current},TVDI_{previous\;one\;year},$ and $TVDI_{previous\;two\;year}$ represent the current, previous one, previous two year TVDIs, respectively. We compared the regression coefficients of the different models to further analyze whether the fitted model increased the fit. The Akaike Information Criterion (AIC) was also calculated to evaluate the benefit of adding the TVDI from the previous year to the regression model between TVDI and WUE. The new model (using two/three years of TVDI data) is considered an improvement over the old model (using one year/two years of TVDI data) if the AIC value is reduced by more than 3.0 [6].

## 3. Results

### 3.1. The Mean WUE in Central Asia

**Figure 1:**
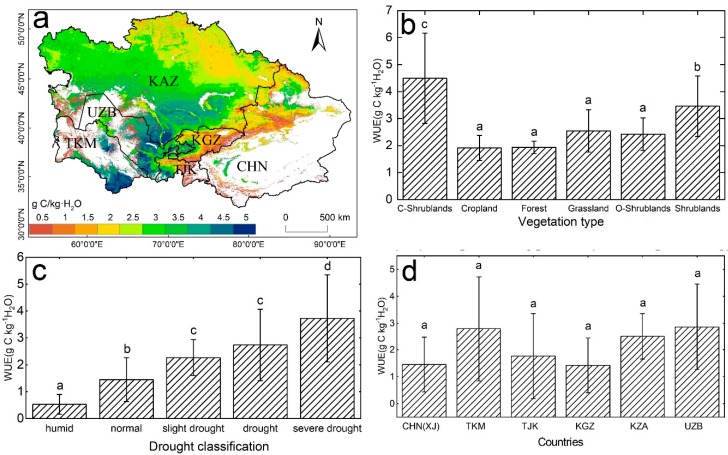
(**a**) Average water use efficiency (WUE) of Central Asia for the period of 2000–2014. White areas represent barren, snow, and water body pixels. Differences in the mean annual WUE among (**b**) vegetation types, (**c**) drought classifications, and (**d**) countries. C-Shrublands represent closed shrublands and O-Shrublands represent open shrublands. Different letters represent significant differences at the 0.05 level.

Overall, the mean GPP in Central Asia showed a high consistency with ET during 2000 to 2014 (Figure S1a,b). The highest GPP and ET were both found in the Ili Valley, Xinjiang; high-altitude mountainous areas of Kyrgyzstan; and mountainous areas in the northeast regions of Kazakhstan. The lowest GPP and ET were observed in some severely arid areas, that is, the margin of the Gurbantunggut Desert in northern Xinjiang, the southern regions of Kazakhstan, and the southeastern regions of Uzbekistan and Turkmenistan. For the period 2000 to 2014, the mean annual WUE in Central Asia was 2.56 g C/kg·H_2_O, which exhibited a remarkable spatial heterogeneity (Figure 1a). Some arid regions in southeastern Uzbekistan and Turkmenistan, and southern Kazakhstan had a high WUE, which ranged from 2.5 to 5.1 g C/kg·H_2_O. The regions with a low WUE (<0.5 g C/kg·H_2_O) were found in the high-altitude mountains in Kyrgyzstan and Tajikistan, and the Tianshan Mountains in Xinjiang. At the biome level (Figure 1b), closed shrublands showed the highest WUE (4.49 g C/kg·H_2_O), and forests and croplands had the lowest WUE. According to the drought classification based on the TVDI (Figure 1c), severe drought ecosystems had the highest WUE (3.73 g C/kg·H_2_O), followed by drought and slight drought ecosystems, and the lowest WUE (0.53 g C/kg·H_2_O) was observed in humid ecosystems. This result is consistent with previous studies, which showed that humid regions are characterized by a low WUE [3,14]. The comparison of the WUE across the different countries revealed that UZB (2.86 g C/kg·H_2_O) and TKM (2.79 g C/kg·H_2_O) had a higher WUE than KZA (2.51 g C/kg·H_2_O), TJK (1.77 g C/kg·H_2_O), CHN (XJ) (1.46 g C/kg·H_2_O), and KGZ (1.43 g C/kg·H_2_O).

### 3.2. The Spatiotemporal Dynamics of WUE and Drought in Central Asia

**Figure 2:**
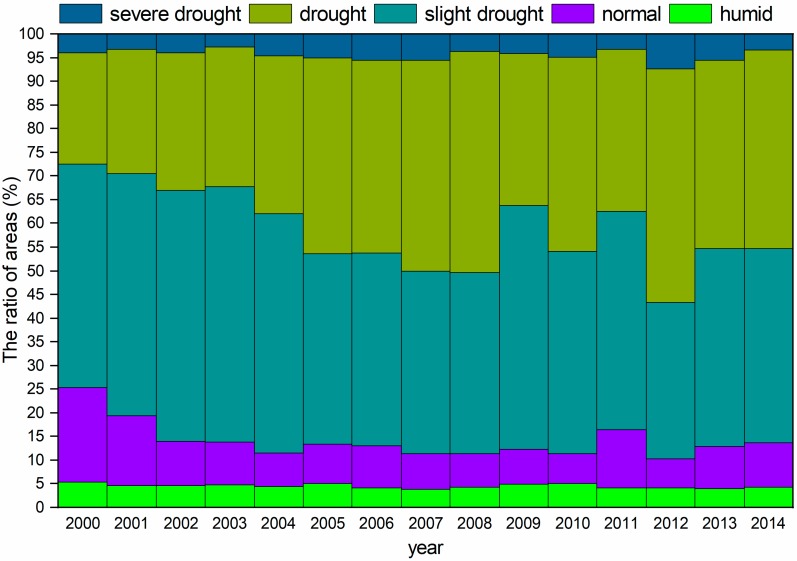
The area ratios of different drought levels from 2000 to 2014.

Figure 2 clearly indicates that arid and slightly arid areas are mainly distributed throughout Central Asia, and these areas cover approximately 80% of the total area. The areas of severe drought and drought showed increasing trends, while the slight drought, normal, and humid areas showed decreasing trends from 2000 to 2014. The area of drought showed a continuously increasing trend from 2000 to 2008, while the areas were relatively small from 2009 to 2011; then, the drought area increased after 2012; the trend of the change in slight drought area was opposite the change in drought area, which suggests that Central Asia experienced a continuous drought from 2000 to 2008. This drought pattern was found in other studies that examined the occurrence of serious drought due to an increase in ET after 2000 [40].

**Figure 3:**
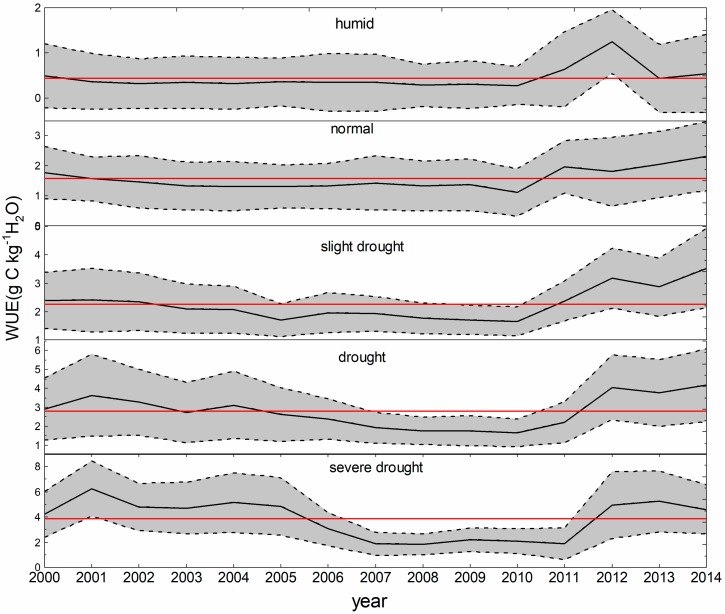
The WUE changes in different drought classifications from 2000 to 2014; the red line is the mean multiyear WUE, and the grayscale represents the standard deviation of WUE.

The WUE changes of different drought classifications over time are shown in Figure 3. Overall, the WUE declined starting in 2000, and the decreasing trend stopped in 2010, except for the severely arid region, where the WUE declined starting in 2001, and the decreasing trend stopped in 2011. The longer duration of the decrease in the WUE in the severely arid region with the subsequent increase in the level of drought results in a lower multiyear mean WUE, which means that the drier conditions in the later years result in a decrease in WUE to the average annual WUE. For example, the WUE was less than the multiyear mean WUE in the humid region (2000), normal region (2000), slight drought region (2002), drought region (2004), and severe drought region (2005).

**Figure 4:**
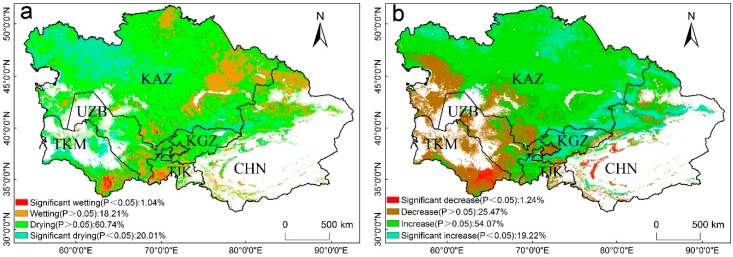
(**a**) Trend of annual temperature vegetation dryness index (TVDI) and (**b**) annual WUE in Central Asia from 2000–2014. White areas represent barren, snow, and water body pixels.

We applied the Mann–Kendall test to determine the trend of WUE and drought over the period of 2000–2014. The spatial distributions of the TVDI trends are shown in Figure 4a. Over the past 15 years, only 1.04% of the vegetated areas in Central Asia exhibited significant wetting. However, 20.01% of the vegetated areas exhibited significant drying trends (*p* < 0.05), and were mainly located in the northwestern part of Kazakhstan and the Tianshan Mountains in Xinjiang; therefore, the significantly arid areas were larger than the significantly wetting areas. Approximately 19.22% of the vegetated areas in Central Asia presented significant increasing trends in WUE, and these areas were mainly located in Kyrgyzstan, the northern region of Xinjiang, and north of Kazakhstan. Only 1.24% of the vegetated areas showed significant decreases in WUE in the south of Xinjiang and southwestern Turkmenistan. The areas where an increase in the WUE was accompanied by drying were observed in Kyrgyzstan and Kazakhstan, except for the northern region of Balkhash Lake. The areas where the drying trends combined with decreasing WUE trends were observed in Turkmenistan and Uzbekistan (Figure 4b). Overall, Central Asia exhibited a drying trend and the WUE increased from 2000 to 2014. Our observations are consistent with those of a previous study, in which Central Asia experienced a serious drought in 2008. The ecosystem WUE decreased linearly, and the TVDI increased rapidly from 2000–2010 (Figure 3). The WUE increased slightly and the TVDI decreased slightly from 2011 to 2014. Therefore, the correlations between the annual WUE and TVDI were divided into two parts in this study, which represent the drought stress period from 2000 to 2010, and the post-drought stress period from 2011 to 2014.

### 3.3. The Relationships Between WUE and TVDI, and Drought Legacy Effects in 2000–2010

**Figure 5:**
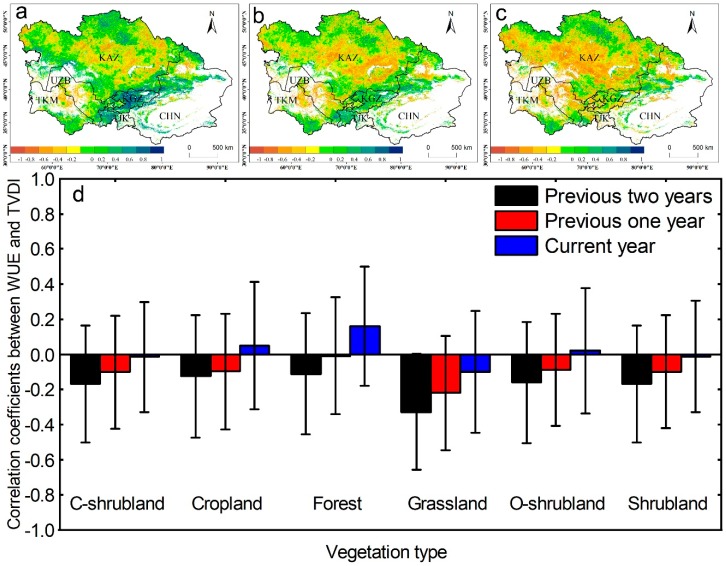
Correlation coefficients between WUE and the TVDI of the (**a**) current year, (**b**) previous one year, and (**c**) previous two years for the period 2000–2010; (**d**) the correlations in different vegetation types. White areas represent barren, snow, and water body pixels.

The ecosystem WUE was relatively low in 2009 and 2010 (Figure 3), although the drought ended in 2008 (Figure 2). To confirm whether drought legacy effects exist in this study, we compared the relationships between the WUE and the TVDI values from the previous one and two years (Figure 5). The areas where the WUE was negatively correlated with the current drought were mainly distributed in Kazakhstan, most of Uzbekistan, and the northwest of Turkmenistan (Figure 5a), and the correlation coefficients between the WUE and the TVDI values from the previous year and previous two years were still negative. The areas where the correlation coefficient between the WUE and drought was positive were located in Tajikistan, Kyrgyzstan, and the Ili region of Xinjiang, and these areas are all located in the Tianshan Mountains. However, when the drought in the first two years was compared to that in the previous year, the correlations in some of these areas changed into negative correlations. In the vegetation classification, the WUE values in C-shrubland, grassland, and shrubland were negatively correlated with drought in the year, and negatively correlated with drought in the previous year and the previous two years. The forest, farmland, and open shrubland areas were positively correlated with the current drought, but there was a negative correlation between the drought in the first two years and that of the previous year.

**Figure 6:**
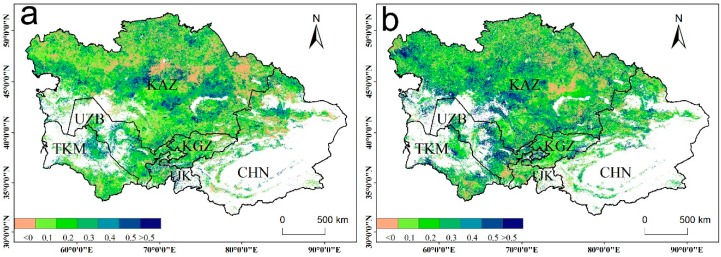
Different model R^2^ values between (**a**) previous one year and the current year, and (**b**) previous two years and previous one year. White areas represent barren, snow, and water body pixels.

**Figure 7:**
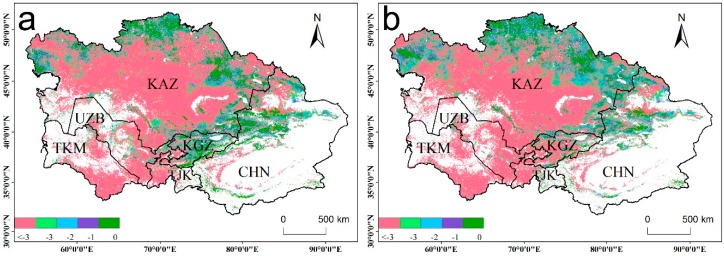
The difference in the Akaike Information Criterion (ΔAIC) value between (**a**) Model 2 and Model 1, and (**b**) Model 3 and Model 2. White areas represent barren, snow, and water body pixels.

To explore the drought legacy effect, we compared the R^2^ value of the models fitted between the current WUE and that in the different years of drought. Figure 6a shows that compared with the R^2^ value of the model fitted to the drought of the current year, the R^2^ value of the model for the previous year increased in most areas of the study area. Similarly, the R^2^ value was also significantly improved for the model fitted to the same two years of drought. To better understand whether the drought in the previous one and two years has a significant impact on the current year WUE, we also compared the AIC values of the different models. Figure 7 shows that the AIC value of Model 2 decreased significantly compared with that of Model 1 in most areas of Central Asia. Similarly, Model 3 exhibited a better performance than Model 2. Therefore, the effects of drought lag exist in our study area, and the current year WUE will be affected by drought in the previous year and the previous two years.

### 3.4. The Relationships Between WUE and TVDI in 2011–2014

**Figure 8:**
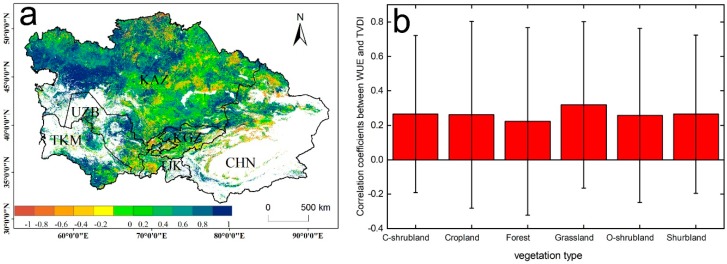
(**a**) Correlation between WUE and TVDI for the period 2011–2014; (**b**) correlations for the different vegetation types. White areas represent barren, snow, and water body pixels.

The correlations between the annual WUE and TVDI showed a large spatial heterogeneity in the vegetated areas of Central Asia from 2011–2014 (Figure 8a,b). The WUE of the ecosystems positively responded to the TVDI in Central Asia, accounting for approximately 66.7% of the vegetated areas, except for the oasis in the southern region of Xinjiang and the middle region of Kazakhstan. This pattern is similar to that reported in certain regional studies, and suggests a divergent response of WUE to drought among different ecosystems (e.g., shrublands and cropland) [6,14]. Compared to the relationship in 2000–2010, all of the vegetation types (cropland, forest, shrublands, and grassland) were positively correlated with the TVDI in 2011–2014 (Figure 8).

### 3.5. Changes in WUE with Shifts in Two Different Drought Periods

**Figure 9:**
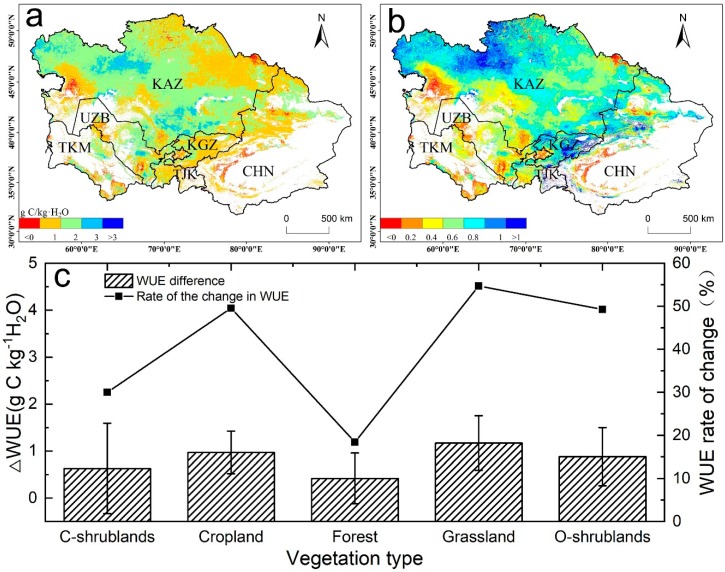
(**a**) Overall changes, (**b**) the rate of change, and (**c**) WUE changes in the different biomes under the transition from dry years (2000–2010) to wet years (2011–2014).

To further investigate how the WUE changed during the two periods, we analyzed the WUE difference between the drought and post-drought periods, which showed that 97.81% of the total vegetation land showed increasing trends (Figure 9). The area with the highest WUE increase rate was located along the Tianshan area and northwestern Kazakhstan. In the post-drought period, the WUE of closed shrublands, cropland, forest, grassland, and open shrublands increased by 30.03%, 49.57%, 18.39%, 54.71%, and 49.28%, respectively, compared with those in the drought period. The results demonstrate that WUE can respond quickly from dry to wet transitions in arid regions. In general, when the ecosystem undergoes a transition from a dry year to a wet year, the ecosystem WUE will exhibit a decreasing trend. However, in this study, we found that the WUE increased as the ecosystem experienced a transition from a dry to humid environment. The occurrence of this phenomenon may be related to the differences in plant adaptation mechanisms and drought intensity.

## 4. Discussion

### 4.1. Mean WUE across Different Vegetation Types

In this study, the low-productivity ecosystems had the highest WUE, such as open shrublands, grasslands, and closed shrublands, while the WUE was low in forests and farmlands (Figure 1). There are two possible reasons for this finding, namely: (1) The areas with high WUE are mainly distributed in Turkmenistan and Uzbekistan, where the vegetation is characterized by relatively low GPP growth in arid regions, and many studies have shown that plant leaf stomatal conductance in arid areas is more sensitive to drought than that in humid areas. Drought promotes the stomatal closure of plant leaves, resulting in a decrease in the rate of evapotranspiration before the photosynthesis rate has decreased [53]. This process led to improvements in the plant WUE. (2) Water restriction is a major feature of arid areas, and studies have shown that the global ET declined in the first decade of the 21st century; moreover, the ET in Central Asia declined as a result of the El Nino effect. The global ET increased significantly from 1982–1997; then, the increasing trend began to slow until the end of 2008 [40]. Microwave remote sensing data also showed a decreasing trend in global soil moisture between 1998 and 2008 [54]. Therefore, the decreasing trend of ET due to soil moisture loss is also the cause of the change in WUE; high WUE with low GPP is caused by low ET in the arid region. High-productivity forests are usually located at high-altitude regions (Tianshan Mountains, Kyrgyzstan, Tajikistan, Xinjiang, and China), which generally have better water conditions; therefore, high temperature environments increase soil moisture evaporation and vegetation transpiration, thereby reducing the WUE in high vegetation areas. Cropland WUE is relatively low compared with that of other vegetation, because most of the crops are usually water-consuming plants [55]. The differences in the spatial distribution of the mean WUE in Central Asia are mainly controlled by water distribution, vegetation type, altitude, and other factors, and this conclusion is consistent with that of other studies [28].

### 4.2. Serious Drought will Lead to the Drought Legacy Effect

In this study, a continuous drying period was found from 2000 to 2008, and then the drought trend eased in 2009 (Figure 2); however, the WUE was still very low in 2009 and 2010. This low WUE was due to the drought legacy effect that was found in a large body of previous studies, which reported that the effects of drought on ecosystem WUE exhibit a legacy effect [6,24,56]. However, most of these studies compared the relationship between WUE and drought in the previous year. In our study, we compared the correlations between the WUE and the drought in the current year, the previous year, and the previous two years (Figure 5), which revealed a similar spatial correlation distribution. Compared to the current drought-related relationship, the correlation between the droughts of the previous two years is high, and the response direction of the WUE is consistent across most vegetation types (Figure 5). This finding indicates that the WUE was affected by the drought in the previous one and two years in most vegetation areas during the drought periods. Previous studies suggested that arid plants respond to a moisture restriction environment by reducing aboveground biomass or even hibernating in consecutive dry years, and the biomass increases rapidly when the moisture is sufficient [56]; however, serious declines in soil moisture cause successive years of drought, and one year of recovery is not sufficient to provide the moisture to meet the basic physiological needs for vegetation growth. This phenomenon may explain the fact that the WUE was still very low in 2009 and 2010, although the drought ended in 2008. Although our study found that the WUE exhibited a significant correlation with the previous one to two years of drought, there is still some uncertainty in the analysis of the legacy effect of drought on the ecosystem, because the legacy effect on the ecosystem may be caused by drought or the confounding effects of other factors, such as radiation intensity, temperature, and human land-use [57,58,59]. Therefore, we should strengthen the distinction between the factors that influence the ecosystem legacy effects in future research.

### 4.3. The Relationship between WUE and Drought in Two Periods

**Figure 10:**
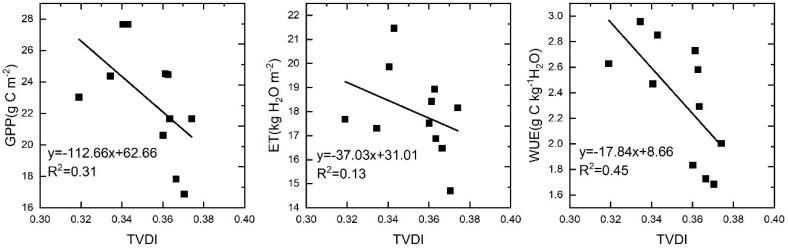
Regression analysis of drought intensity and gross primary productivity (GPP), evapotranspiration (ET), and WUE.

In this study, we found that the response of WUE to drought was the opposite in drought periods (2000–2010) and post-drought periods (2011–2014). In the different vegetation types, negative correlations were mainly found in grasslands and closed shrublands during drought periods. Previous studies have shown that WUE has a negative correlation with drought in arid ecosystems, but the main reason for these results was that ET is more sensitive to hydrological climate change than GPP, and the decline in ET to values lower than GPP led to a negative correlation between WUE and drought in arid areas [60]. Other studies suggested that the response of the ecosystem WUE to drought varies with drought intensity, duration, and vegetation type [14]. In this study, GPP, ET, and WUE all exhibited a negative correlation with drought (Figure 10). GPP was more sensitive to drought than ET, which was the main reason for the negative correlation between the WUE and drought. Grassland was the main vegetation type with a negative correlation. Grassland is usually highly sensitive to water utilization, although grassland has the ability to survive after biomass losses, because of long-term adaptation. However, this ability is based on the intensity and duration of the drought. Therefore, the decline in GPP due to persistent drought is the main reason for the negative correlation between WUE and drought in this study. The positive correlation between ecosystem WUE and drought in the post-drought periods suggests that vegetation gained resilience after obtaining moisture. Therefore, when studying the relationship between WUE and drought, it is important to determine whether the study area experienced drought first, because WUE was found to be negatively correlated with drought in the drought period, while WUE and drought showed a positive correlation after the drought event in this study.

### 4.4. The WUE Changes in Drought and Post-Drought Periods

The stability of ecosystems is related to not only the resistance of plants to water stress, but also the rate of recovery in the post-drought period, which is also referred to as ecosystem resilience. Ecosystem resilience is affected by a number of factors, including the severity and duration of water deficiencies, vegetation types [61], types of drought damage [62], plant growth rate and competition among plant species [63], and the differences in drought observational scale. In this study, the WUE declined during drought periods, revealing a weak resistance to drought in arid ecosystems, which is also likely to be a survival strategy for plants under dry conditions. After the drought event, the ecosystem is highly sensitive to abrupt changes in the environment, and the increase in WUE in the various vegetation types also indicated the resilience of the arid ecosystem, which is consistent with previous studies [64]. Ecosystem WUE is a common connection between the capabilities of biomes and ecosystems, which adapt to changing hydrologic climate conditions by regulating their WUE. In arid areas, ecosystems can self-regulate to restore themselves to pre-drought conditions after severe and persistent drought once the water conditions become appropriate. This study comprehensively analyzes the rapid recovery capacity of arid ecosystems and further confirms the survival strategies of arid ecosystems to cope with drought.

Some results of this study have also been found in previous studies, such as the spatial patterns of the WUE across the different drought levels and biome types; for example, the ecosystem WUE exhibited a rapid increase after the drought event. However, there are also some differences with previous studies. For example, the drought legacy effect shows that severe drought will increase the impact on ecosystems. The main reason for this difference may be the divergence of drought intensity in different studies. The relationship between WUE and drought found in this study is different from that in previous studies, which may be because the study period in this study was divided into two stages, namely: the drought period (2000–2010) and the post-drought period (2011–2014). The response of the WUE to drought can provide a theoretical basis for understanding ecosystem responses in arid regions under future climate change. Thus, in future studies, the research time should be lengthened and field data should be added to improve the accuracy of remote sensing data in the future.

## 5. Conclusions

Our study shows that high and low multiyear mean WUE values were mainly observed in arid and mountainous regions, respectively. Overall, Central Asia exhibited a dry trend, and the WUE exhibited an increasing trend from 2000 to 2014. Different responses of the ecosystem WUE to drought were identified in the two periods (drought stress period from 2000 to 2010 and the post-drought stress period from 2011 to 2014), in which the WUE showed a negative response to drought in the drought period, and positive relationships between the WUE and drought were found in the post-drought period. Serious droughts led to significant legacy effects, in which the ecosystem WUE was affected by the previous two years of drought. In addition, the WUE increased rapidly from the drought to the post-drought period, indicating that arid ecosystems have resilience. As expected from the current climate change scenarios, drought will become more frequent and severe in most areas of the world. Our research results can provide a certain reference for understanding how ecosystems will respond to climate change. To accurately predict the time and intensity of drought, future research should make full use of existing data to investigate the changes in ecosystems before drought events as well as the drought thresholds that may cause ecosystems to collapse.

## Supplementary Materials

The following are available online at https://www.mdpi.com/1424-8220/20/3/581/s1, Figure S1: Average GPP and ET of Central Asia for the period 2000–2014.
