# Supplementary material for: Assessing the Response of Ecosystem Water Use Efficiency to Drought During and after Drought Events across Central Asia
Source: Sensors (Basel). 2020 Jan 21;20(3):581. doi: 10.3390/s20030581 (PMC7038223; doi:10.3390/s20030581)
Supplement: Supplementary file 1 [file sensors-20-00581-s001.pdf]

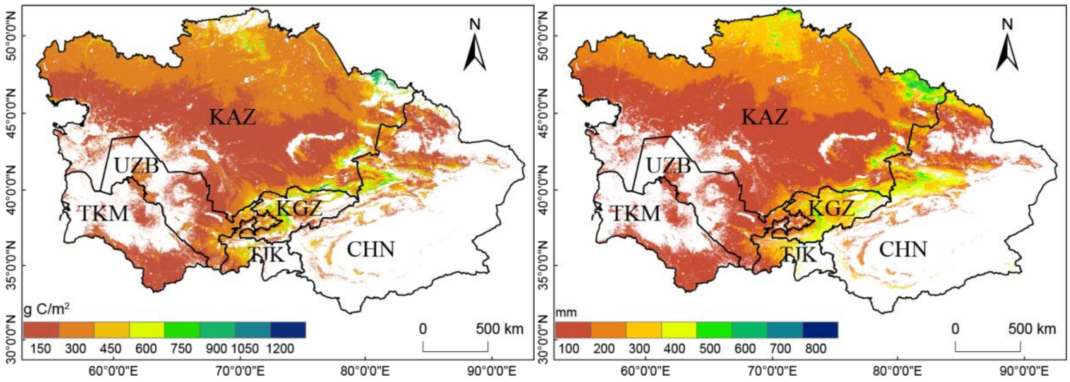

Figure S1. (**a**) Average GPP; (**b**) average ET of Central Asia for the period 2000-2014; white areas represent barren, snow and water body pixels.
